# Pathogenesis of Anti-melanoma Differentiation-Associated Gene 5 Antibody-Positive Dermatomyositis: A Concise Review With an Emphasis on Type I Interferon System

**DOI:** 10.3389/fmed.2021.833114

**Published:** 2022-01-24

**Authors:** Huifang Hu, Hang Yang, Yi Liu, Bing Yan

**Affiliations:** Department of Rheumatology and Immunology, Rare Diseases Center, Frontiers Science Center for Disease-Related Molecular Network, Institute of Immunology and Inflammation, West China Hospital, Sichuan University, Chengdu, China

**Keywords:** anti-MDA5 antibody, MDA5^+^ DM, CADM, type I IFN, IFN I system, pathogenesis

## Abstract

Anti-melanoma differentiation-associated gene 5 antibody-positive dermatomyositis (MDA5^+^ DM) is typically characterized by cutaneous manifestations, amyopathic or hypomyopathic muscle involvement, and a high incidence of rapid progressive interstitial lung disease (RP-ILD). However, the exact etiology and pathogenesis of this condition has yet to be fully elucidated. Melanoma differentiation-associated gene 5 (MDA5), as the autoantigen target, is a member of the retinoic acid-inducible gene-I (RIG-I) family. The MDA5 protein can function as a cytosolic sensor that recognizes viral double-strand RNA and then triggers the transcription of genes encoding type I interferon (IFN). Therefore, it was presumed that viruses might trigger the overproduction of type I IFN, thus contributing to the development of MDA5^+^ DM. Emerging evidence provides further support to this hypothesis: the increased serum IFNα level was detected in the patients with MDA5^+^ DM, and the type I IFN gene signature was upregulated in both the peripheral blood mononuclear cells (PBMCs) and the skin tissues from these patients. In particular, RNA sequencing revealed the over-expression of the type I IFN genes in blood vessels from MDA5^+^ DM patients. In addition, Janus kinase (JAK) inhibitors achieved the promising therapeutic effects in cases with interstitial lung disease (ILD) associated with MDA5^+^ DM. In this review, we discuss the role of the type I IFN system in the pathogenesis of MDA5^+^ DM.

## Introduction

Dermatomyositis (DM) is a form of idiopathic inflammatory myopathy (IIM) that is characterized by chronic systemic inflammation and predominantly affects the skeletal muscle, skin, joints, and lungs. According to the different myositis-specific autoantibodies, there are several highly heterogeneous DM phenotypes (1, 2). In particular, patients with anti-melanoma differentiation-associated gene 5 (MDA5) autoantibodies had the typical rashes of DM, but the mild or even absent muscle involvement, and an increased risk of rapid progressive interstitial lung disease (RP-ILD), a condition that often leads to high mortality (3–5). According to previous studies, the mortality of RP-ILD related to anti-melanoma differentiation-associated gene 5 antibody-positive dermatomyositis (MDA5^+^ DM) in the first year ranged from 34.8% - 80% (3, 6, 7).

The exact pathogenesis of MDA5^+^ DM remains largely unknown, although the interaction of environmental factors and genetic factors are generally considered as the initiating event (8). Recently, more emerging evidence has suggested that type I interferon (IFN) could play a critical role in MDA5^+^ DM. Thus, we summarize the current evidence regarding the role of IFN I system in MDA5^+^ DM.

## MDA5 and the IFN I System

### The Function of MDA5

The anti-MDA5 antibody was first discovered in a Japanese patient cohort with clinically amyopathic dermatomyositis (CADM) in 2005 (9). Subsequent studies demonstrated that MDA5, the autoantigen target, was coded by the interferon-induced helicase C domain-containing protein 1 (IFIH1) gene. MDA5, as a member of the retinoic acid-inducible gene-I (RIG-I) family (10–14), can recognize viral dsRNA (14–16). After activation, MDA5 binds to an adaptor, mitochondrial antiviral signaling protein (MAVS), on the outer membrane of mitochondria, and then activates the interferon regulatory family 3 (IRF3) via tumor necrosis factor receptor-associated factor 3 (TRAF3) and TANK-binding kinase 1 (TBK1) (10, 14, 16–19). Finally, these events induce the production of enormous quantities of type I IFNs, thus causing antiviral properties (20–22).

### The IFN I System

Type I IFNs are polypeptides that exhibit antiviral properties and were first described over 60 years ago (23). Subsequently, these polypeptides were found to play the critical roles in inflammation, cancer, and autoimmune diseases (10, 21, 24). Generally, the type I IFN system is broadly defined as the type I IFNs themselves, along with their inducers, cells, and other molecules involved in the pathways that lead to the production and functional effects of type I IFNs (25). In humans, type I IFNs predominantly consist of IFNα and IFNβ, although there are less well known subtypes, including IFNω, IFNκ, and IFNε. Almost all nucleated cells are capable of producing IFNβ, while plasmacytoid dendritic cells (pDCs) are the predominant producers of IFNα in most situations (26). The production of type I IFN is mediated by toll-like receptors (TLRs) (TLR-dependent) or other systems, such as RIG-I and MDA5 (TLR-independent), depending on the nature of the inducers involved (10, 21). TLR3/7/8/9, the endosomal membrane receptors expressed in many innate immune cells, are the predominant TLRs in the TLR-dependent pathways. In particular, TLR7/9 are expressed preferentially in the endosomal membranes of pDCs (21, 22).

IFNα/β bind to the IFNα receptor (IFNAR, composed of IFNAR1 and IFNAR2 subunits) thereby causing activation of receptor-associated tyrosine kinases like Janus kinase 1/2 (JAK1/2) and tyrosine kinase 2 (TYK2). Then, JAK1 and TYK2 can phosphorylate the transcription factors known as signal transducer and activator of transcription 1 (STAT1) and STAT2. Phosphorylated STAT1 and STAT2 dimerize and translocate to the nucleus where they form a trimolecular complex referred to as IFN-stimulated gene factor 3 (ISGF3) with IFN-regulatory factor 9 (IRF9) (13, 24, 27). This complex binds specific elements known as IFN-stimulated response elements (ISREs). This binding initiates the transcription of IFN-stimulated genes (ISGs), such as Mx1, IFIT2, and ISG15 (28, 29). The expression of ISGs, the so-called IFN signature, can target the distinct steps in the viral replication cycle (30). Type I interferon signaling is tightly regulated by various pathways such as post-translational modification of signaling molecules and epigenetic modification of gene expression. Aberrant IFN signaling is widely associated with pathological conditions such as autoimmune diseases, chronic infection and cancer (13).

Except direct antiviral properties, type I IFN augments the innate and adaptive response by enhancing the cytotoxic effect of natural killer (NK) cells and intracellular killing by macrophages, stimulating dendritic cells (DCs) to mature into effective antigen presenting cells (APCs), and increasing expression of class I and II major histocompatibility complex (MHC). Type I IFNs activate a number of immune cells including macrophages, DCs and T cells. DCs matured and stimulated by IFNα/β express co-stimulatory molecules (e.g., CD80 and CD86), produce chemokines (e.g., CXCL9 and CXCL10) and cytokines (e.g., IL-15), and mount an enhanced antiviral immune reaction. Thus, the effector function of T and B cells are promoted by type I IFN. Subsequently, type I IFN can promote immunoglobulin (Ig) class switching and antibody production. Meanwhile the generation of memory cells is augmented (26, 31).

## Footprints of IFN I System in Organs Involved in MDA5^+^ DM

### Skin

Individuals with MDA5^+^ DM have a distinctive mucocutaneous phenotype, involving cutaneous ulceration and palmar papules (4, 32, 33). Interestingly, IFNκ, which is highly expressed in keratinocytes, has been shown to expressed at high levels in the skin of patients with MDA5^+^ DM (34, 35). A previous study showed that IFNκ enhanced IFN responses and photosensitivity in the pathological progression of cutaneous lupus erythematosus (36). Therefore, the potential role of keratinocytes and IFNκ in the skin of patients with MDA5^+^ DM might be more substantial than that of IFNα. Histological analysis of the skin tissues from these patients revealed the significant perivascular inflammation and Mx1 expression in the blood vessels of the superficial and deep dermis (37). Skin IFN I transcriptomic signature analysis in three MDA5^+^ DM patients further found that FIT2C, CXCL10, and IFIH1, were the most highly upregulated genes in the skin tissue (35).

The pathogenic mechanism underlying cutaneous ulcers in MDA5^+^ DM is known to involve vasculopathy. Pathological evidence for vasculopathy has been found in the biopsies of cutaneous ulcers from patients with MDA5^+^ DM (33, 38). Biomarkers related to endothelial damage, including serum soluble intercellular adhesion molecule-1 (sICAM-1), soluble vascular cell adhesion molecule-1 (sVCAM-1), endothelin-1 (ET-1), and von Willebrand factor (vWF), were found to be expressed at high levels in patients with MDA5^+^ DM. Furthermore, the circulating levels of ET-1 were positively correlated with the levels of Galectin-9 (Gal-9) in MDA5^+^ DM patients (39). Gal-9 mRNA expression were positively correlated with the mRNA levels of IFN-I inducible genes in PBMCs from MDA5^+^ DM patients (40). Collectively, IFN I system may be suggested to be involved in the skin damage in patients with MDA5^+^ DM.

### Muscle

The clinical signs of myositis are frequently mild or absent in patients with MDA5^+^ DM (3, 4, 6). In a previous study, Allenbach et al. analyzed the expression of six ISGs in muscle, including OAS1, ISG15, OAS3, Mx1, RIG-I, and IFIH1. Although the IFN signature was up-regulated in groups of patients with MDA5^+^ DM and classic DM when compared with normal muscle, the up-regulation of ISGs was less significantly in MDA5^+^ DM patients than that in classic DM patients (41). A study from China investigated the mitochondrial changes and MAVS-type I IFN signaling pathways in the muscles of MDA5^+^ DM patients (42). These authors found that the IFN-I inducible proteins (MDA5, MAVS, IRF7, and ISG15) were overexpressed in the muscles of MDA5^+^ DM patients, suggesting that the MAVS-type I IFN pathway is broadly involved in the muscle pathology of MDA5^+^ DM.

### Lung

ILD is the most striking systemic complication in patients with MDA5^+^ DM and could present as worsening respiratory distress, hypoxemia, and radiographic deterioration over the course of days or weeks (4). A recent study demonstrated that serum levels of IFNα and ferritin were significantly higher in MDA5^+^ DM patients, and serum IFNα could be used as a biomarker for MDA5^+^ DM patients with RP-ILD (43).

The significance of anti-MDA5 antibody in MDA5^+^ DM may represent another piece of important evidence for the involvement of IFN I system in this disorder. A recent study revealed that RNA-containing immune complexes (ICs) formed by MDA5 and anti-MDA5 antibody could stimulate IFNα production *in vitro*. Further co-immunoprecipitation verified the interaction between ICs and TLR7. Confocal images also showed co-localization of ICs with TLR7, thus suggesting that the MDA5 ICs could induce the production of IFNα via TLR7 (44). A growing body of data now suggests that the level of anti-MDA5 antibody correlates with disease activity and prognosis (32, 45, 46). Furthermore, Lian X et al. proposed the FLAIR risk score model, consisting of ferritin, lactate dehydrogenase, anti-MDA5 antibody, high-resolution CT imaging score, and RP-ILD, to predict the prognosis in patients with CADM-ILD. In this model, high titers of anti-MDA5 antibody were considered to be one of the weighting factors, which suggests a poor prognosis (47).

Downstream chemokines and cytokines of IFN I system are known to be upregulated in MDA5^+^ DM patients with ILD. In a hierarchical cluster analysis of cytokine profiles, levels of IFN-related cytokines (IFNγ, IFNα, and IP-10, an IFN-induced proteins) were significantly higher in the anti-MDA5 antibody-positive subgroup patients than in the anti-TIF1γ antibody-positive subgroup patients (48). Elevated levels of CX3CL1 were found in ILD patients associated with MDA5^+^ DM and were correlated with the titers of the anti-MDA5 antibodies (49, 50). Type I IFN could induce CX3CL1 expression in pulmonary vascular endothelial cells (51). In a previous study, CX3CL1 level was correlated to CD8^+^ T cells counts and the severity of lung parenchyma impairment (52). CX3CL1 was reported to enhance endothelial transmigration of non-classical monocytes in the lungs of patients with ILD, the recruitment of M2 macrophages, thereby promoting local pulmonary fibrosis (53, 54).

Given the role of the type I IFN pathway in MDA5^+^ DM, it is reasonable that JAK inhibitors may represent a potential therapeutic option for ILD complications. Five refractory RP-ILD cases associated with MDA5^+^ DM received a combination therapy including tofacitinib and achieved a significant higher survival rate (55). Further, in early-stage MDA5^+^ DM-ILD patients who received JAK inhibitors, a significantly increased survival rate was observed (56). Another study described two refractory JDM patients with anti-MDA5 antibodies who were treated with a JAK inhibitor (tofacitinib). Both patients demonstrated a significant reduction in the IFN score and improvement in disease activity (57). However, this treatment effect needs to be investigated further.

## The Mechanisms Underlying IFN I Pathway in MDA5^+^ DM

As mentioned above, type I IFN system may be over-activated in MDA5^+^ DM. However, the exact underlying mechanisms has yet to be elucidated. We propose that environmental factors may trigger the aberrant production of type I IFN in genetically susceptible individuals. This process will subsequently induce type I IFN-meditated cellular pathology.

### Environmental and Genetic Factors

Viral infections are speculated to be an initiating factor in the development of MDA5^+^ DM. RNA viruses, such as the coxsackie B virus, parvovirus B19, and hepatitis C virus, have been reported to be involved in myositis (58–60), all of which could activate MDA5 (14). Recently, two independent studies have suggested that environmental factors can influence the onset of MDA5^+^ DM: One epidemiological study in Japan showed that the presence of anti-MDA5 antibody in CADM patients was negatively associated with the population size of their city (61). The other study reported that patients with MDA5^+^ DM-ILD were mainly identified between October and March in individuals residing near freshwater (62).

With regards to genetic risk factors, frequencies of HLA-DRB1^*^0401 and ^*^1202 were significantly higher in a cohort of Chinese Han subjects with MDA5^+^ PM/DM (63), while HLA-DRB1^*^0101 and ^*^0405 were associated with susceptibility to this disorder in the Japanese population (64). Ethnic differences may have an impact on the propensity of this disease, in spite of no association between MDA5^+^ DM and the HLA allele region analyzed in the Caucasian population (65). Interestingly, a genome-wide association study of 592 patients with IIM revealed a splice variant of the WDYF4 gene in CADM patients. This variant and tr-WDFY4 (a truncated isoform of WDFY4) were shown to bind TLR3, TLR4, TLR9, and MDA5 and could enhance activation of the NF-κB pathway (66).

### IFN I-Meditated Cellular Pathology

Type I IFN can function as the central liaison between the innate and adaptive immune systems, which is the essential player in autoimmune diseases.

Type I IFN can exert direct influence on T cells. Peripheral blood lymphocytes are often reduced when serum levels of IFN increased. A previous study showed that a significantly higher proportion of MDA5^+^ DM patients had reduced peripheral CD4^+^ and CD8^+^ T cell counts (46). A more recent study revealed that CD4^+^ CXCR4^+^ T cells were increased in PBMCs and bronchoalveolar lavage fluid in patients with IIM-ILD. Patients with high CD4^+^ CXCR4^+^ (≥30%) were more likely to be positive for the anti-MDA5 antibody. *In vitro*, peripheral CD4^+^ CXCR4^+^ T cells produced high levels of IL-21, which were found to meditate the proliferation of pulmonary fibroblasts and were inhibited by a JAK inhibitor (67).

Type I IFN could induce DCs to produce B cell activating factor (BAFF), which plays a critical role in B cell survival and Ig class switching (31, 68). Notably, the upregulation of BAFF following viral infection is IFN-dependent. Monocytes have been shown to release BAFF in response to IFN treatment (68, 69). Matsushita et al. revealed that MDA5^+^ DM patients exhibited increased levels of BAFF when compared with healthy controls. Furthermore, serum BAFF levels in MDA5^+^ DM patients were reduced following immunosuppressive therapy along with the levels of anti-MDA5 antibody and ferritin (70). In addition, Zhang et al. found that IFNα was correlated to BAFF in patients with MDA5^+^ DM and that increased levels of BAFF were associated with higher concentrations of Krebs von den Lungen-6 (KL-6) (71), a potential biomarker that reflects the severity of connective tissue disease-associated ILD (72). It has been shown that an excess of BAFF may contribute to the autoreactive B cells survival, thereby causing autoimmune diseases (73).

Although the pathogenicity of the anti-MDA5 autoantibody has not to be fully elucidated, there are two lines of evidence which are shedding the light: One is that RNA-containing ICs, formed by MDA5 and anti-MDA5 antibody, may enhance type I IFN self-circulation as endogenous inducers. A recent Chinese study revealed that MDA5 ICs, in patients with either DM or SLE, could activate pDCs and stimulate the production of IFNα *in vitro*. These authors also confirmed that MDA5 ICs interact with TLR7 to induce the production of IFNα (44). The other is that IgA and IgG are shown to be the major isotypes of MDA5 autoantibodies in patients with MDA5^+^ DM. One study showed that DM/CADM patients with anti-MDA5 IgG1 subclasses were correlated to significantly higher occurrence rates of acute interstitial pneumonia, serum levels of lactate dehydrogenase (LDH), and ferritin. Higher mortality rates were detected in patients who were positive for anti-MDA5 IgG1 and IgG4, thus suggesting that these two IgG subtypes might serve as useful biomarkers for predicting mortality in patients with DM-ILD (74).

Type I IFN also induces the development of autoinflammation (14, 75). Monocytes and macrophages are both essential parts of this process. A previous case report detected macrophages in the alveoli, bone marrow, liver, and spleen of a MDA5^+^ DM patient with RP-ILD and hyper-ferritin (76). In addition, levels of serum soluble CD206, a marker of alternatively activated macrophages, were increased in ILD patients with MDA5^+^ DM and were associated with the poor prognosis (77). In this study, the authors observed the marked accumulation of CD206^+^ macrophages infiltrated into the airspace of a fatal DM-ILD case compared with a surviving patient with CADM-ILD and an early lung cancer patient, thus suggesting the potential role of macrophages in the disease progression of MDA5^+^ DM. As discussed above, type I IFN can induce the expression of CX3CL1 in pulmonary vascular endothelial cells (51). Further studies also verified that CX3CL1 could drive the migration of non-classical monocytes into lung tissue, thereby meditating local fibrotic process (54). An integrated miRNA-mRNA association analysis using circulating monocytes from MDA5^+^ DM patients identified ILD, IFNβ, TLR3, TLR7, TLR9, and the Spi-1 proto-oncogene encoding PU.1 (a transcription factor regulating the differentiation of monocytes), as upstream regulators of MDA5^+^ DM (78). These authors also found that activated monocytes/macrophages contributed to cytokine storms in patients with MDA5^+^ DM with ILD (78). On the other hand, serum levels of C-C motif ligand 2 (CCL2, a chemokine that is also referred to as monocyte chemoattractant protein-1) and IFNβ, were both reduced following the treatment of survivors with MDA5^+^ DM with ILD. It has been suggested that CCL2 guides monocytes along a chemokine gradient to the location of inflammation, such as the lungs (79, 80).

**Figure 1:**
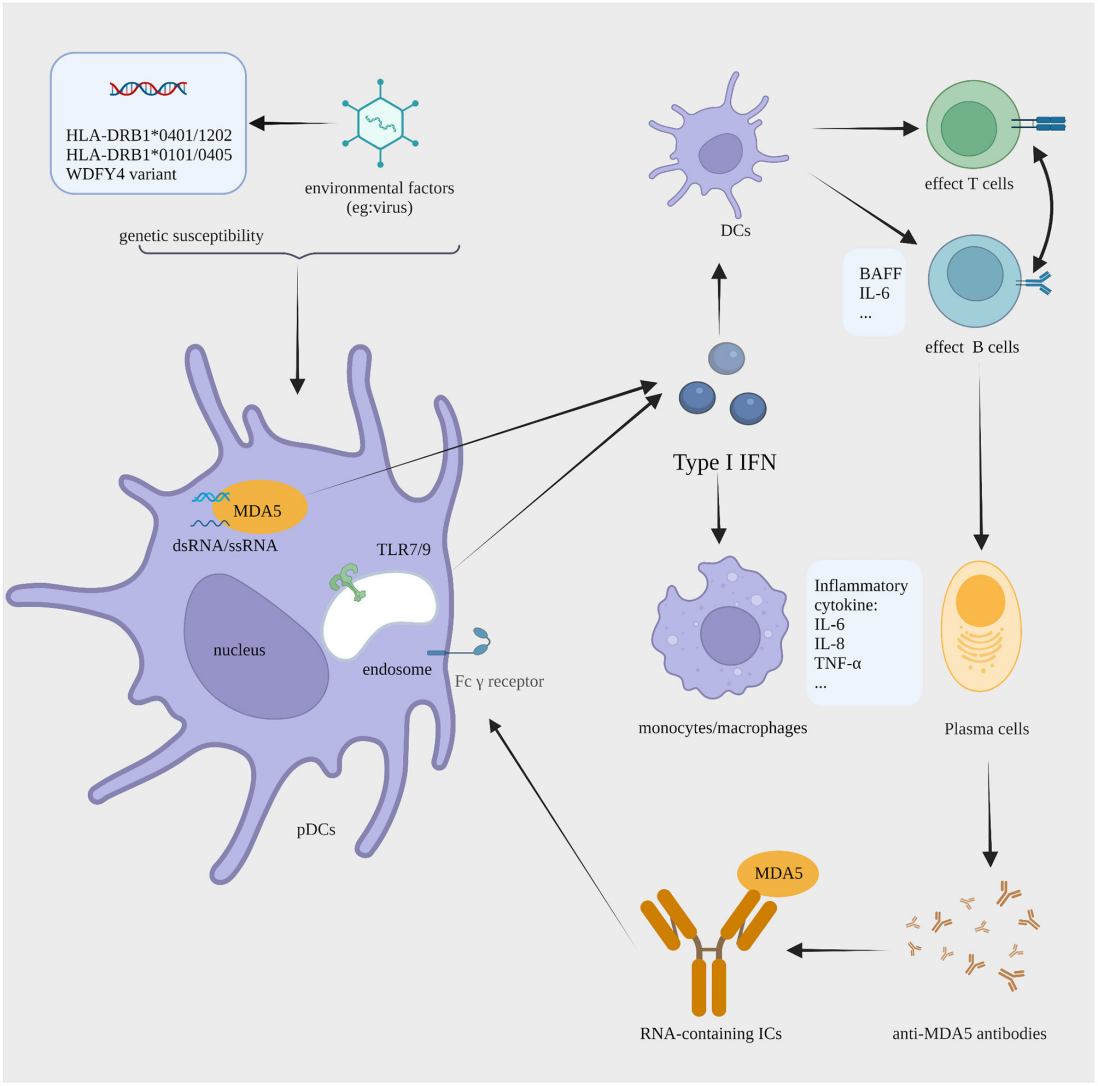
The proposed IFN I-mediated cellular pathology in MDA5^+^ DM. In individuals with a background of susceptibility genes (HLA-DRB1*0101 and *0405, HLA-DRB1*0401 and *1202, or a variant in WDFY4), the dsRNA of unknown viral trigger is sensed by MDA5, causing the aberrant production of type I IFN by pDCs. Type I IFN enhances the antigen presentation of DCs, the effector function of T and B cells, and antibody production by plasma cells. Anti-MDA5 antibodies are generated in large amounts and then recognize MDA5, forming RNA-containing ICs. The ICs can activate type I IFN production via TLR7. All this promotes continued type I IFN production that sustains pathogenesis by a process with features of a vicious circle. Moreover, monocyte and macrophage may contribute to the development of RP-ILD. IFN, interferon; MDA5^+^ DM, anti-melanoma differentiation-associated gene 5 antibody-positive dermatomyositis; pDCs, plasmacytoid dendritic cells; DCs, dendritic cells; ICs, immune complexes; TLR, toll-like receptor; RP-ILD, rapid progressive interstitial lung disease. The figure is created with BioRender.com.

Above all, we make up a jigsaw puzzle of MDA5^+^ DM pathogenesis with an emphasis on the role of type I IFN (Figure 1). In genetically susceptible individuals, unknown environmental triggers are recognized by the MDA5 protein. This process leads to dysregulation in the type I IFN system. As the liaison between the innate and adaptive immune response, the overproduction of type I IFN promotes the antigen presentation of DCs. In addition, type I IFN activates the differentiation of T cells and B cells. Then, pathogenic autoantibodies are generated by plasma cells. RNA-containing ICs formed by MDA5 and the anti-MDA5 antibody might act as the endogenous inducers for type I IFN, thus aggravating the vicious circle of type I IFN system overactivation. Furthermore, the activation of monocytes and macrophages might contribute to the development of RP-ILD.

## Discussion

Accumulating evidence over the past few years has revealed that the dysregulation type I IFN system could play the role in the pathogenesis of MDA5^+^ DM. Given the role of the type I IFN system in MDA5^+^ DM, therapies that target the type I IFN pathway, such as JAK inhibitors, have shown potential therapeutic effect. Except for JAK inhibitors, sifalimumab, one of the anti-IFNα monoclonal antibodies, has shown a target neutralization of a type I IFN signature in the blood of DM and PM patients in a phase Ib clinical trial (81). Thus, anti-IFNα therapies and other new targets of type I IFN pathway would be new treatment options.

## Author Contributions

HH and HY drafted the manuscript and figure. YL and BY supervised the work and revised the manuscript. All authors contributed to the article and approved the submitted version.

## Funding

This research was funded by grant from the National Natural Science Foundation of China (No. 81172870), 1·3·5 project for disciplines of excellence, West China Hospital, Sichuan University (No. ZYGD18015), 1·3·5 project for disciplines of excellence, West China Hospital, Sichuan University (No. ZYJC18003), and the Science and Technology Innovation and Entrepreneurship Seedling Project of Sichuan Province (No. 2019JDRC0101).

## Conflict of Interest

The authors declare that the research was conducted in the absence of any commercial or financial relationships that could be construed as a potential conflict of interest.

## Publisher's Note

All claims expressed in this article are solely those of the authors and do not necessarily represent those of their affiliated organizations, or those of the publisher, the editors and the reviewers. Any product that may be evaluated in this article, or claim that may be made by its manufacturer, is not guaranteed or endorsed by the publisher.
